# The Voluntary Aid Detachments

**Published:** 1910-07-23

**Authors:** 


② The Hospital

A Journal of

The Medical Sciences and Hospital Administration.

New Series. No. 180, Vol. VII. [No. 1252, Vol. XLVIII.] SATURDAY, JULY 23, 1910

### The Voluntary Aid Detachments.

The Chairman and Committee of the County of London Branch of the British Red Cross Society have sent to the offices of The Hospital a quantity of literature dealing with the work which the Society is undertaking in connection with the Voluntary Aid Detachments of the Territorial Force. Those who have followed the articles which have appeared in these columns from time to time on the organisation of the Royal Army Medical Corps (Territorial) will remember that in Mr. Haldane's scheme a gap exists between the Field Ambulances which follow the troops in the firing-line and pick up the wounded on the battle-field, and the General Hospitals, which are to be situated in large towns. The work of transferring the sick and wounded from the Field Ambulances, which must of necessity evacuate their patients rapidly in order to follow the combatant troops in the field, to the General Hospitals is assigned to Voluntary Aid Detachments; and the County Associations are charged with the duty of raising these as best they may. Now the County Associations have their hands a good deal more than full of administrative work which is defined and financially provided for—often very inadequately—by the regulations. It is natural enough, therefore, that they should welcome eagerly the chance of delegating to other bodies the burdensome task of raising and organising this very important part of the medical service of the Army for which no funds at all are provided, and that they should take advantage of the permission given to make all possible use of pre-existing societies. The only two bodies capable of thus helping the County Associations out of the difficulty in which the War Office has placed them in this matter are the St. John Ambulance Association and the British Red Cross Society. The former of these is practically a part of the Order of St. John of Jerusalem in England, and is indeed the principal *raison d'être* for the continued existence of that body. The latter is a society which has not yet celebrated its fifth birthday, and its London branch was started as lately as December last. Notwithstanding its youth, the Red Cross Society has plunged into the work with a light heart, whereas it would appear that the St. John Ambulance Association wanted to see rather more clearly what it was undertaking before committing itself in the same way. This at least is the inference which is suggested by a study of the questions asked in the House of Lords on June 16 by the Earl of Dartmouth, and answered by the Under Secretary for War, Lord Lucas. For out of the ambiguities with which the subject was surrounded it emerged that the St. John Ambulance Association has declined any further to be formally connected with the scheme, whereas at one time it had consented to undertake the work of instructing members of the Voluntary Aid Detachments in first aid and nursing.

In the documents to which reference has already been made is set forth what the Red Cross Society—and particularly its London branch—has undertaken to do. So various are the functions which Voluntary Aid Detachments may be called upon to assume in war time that it is not the least surprising to find that appeals for subscriptions are being widely circulated. For one guinea *per annum* membership can be secured; for five shillings associateship; and those who contribute even smaller sums are to be denominated adherents. All these grades are distinct from the further status of an assistant or of an enrolled member of a Voluntary Aid Detachment. The plain fact of the matter is that an integral part of the Territorial scheme is left entirely unprovided for by the Treasury and the War Office; that the County Associations, already harassed to the verge of distraction by both those Offices, are only too glad to shift responsibility on to any shoulders willing to accept it; and that the British Red Cross Society, which rushed in where the wealthier and warier St. John Association feared to tread, is now confronted with the task of making bricks without straw. In a word, the nation is trying to run its Army not only on the cheap, which is foolish enough in all conscience, but actually (in this respect) for nothing at all; and private benevolence is invoked to make good the deficit. One sentence from the official Red Cross leaflet is enough to show how matters trend: "All expenses must be kept as low as possible, and must be defrayed out of the funds collected.'' Yet in spite of this there is talk of providing hospital ships, hospital trains, and all sorts of expensive luxuries of this sort which should, of course, be a charge upon the national exchequer; while at the same time, to quote another document, "the Red Cross branch of London has adopted the wise precaution of not incurring heavy expense in buying and storing supplies and material in peace. Such a course would only result in deterioration and needlessly recurring expenditure." We admire the valour with which the Red Cross Society has taken up this enormous work, but we admire even more the discretion of the St. John's Council in withdrawing from participation in a scheme which is founded on entirely wrong principles and is bound to be either a failure or a sham. We honour the patriotism of those who are sacrificing time and money on this work, but we feel that disillusionment awaits them. To use an expressive vulgarism, the Red Cross Society seems to have bitten off more than it can chew; and the worst of it is that the British public will ignore this fairly evident fact and rest content in its customary indifference to one of the gravest of the many weak spots in its civilian army of defence as at present constituted.

